# Phenotyping of light-activated neurons in the mouse SCN based on the expression of FOS and EGR1

**DOI:** 10.3389/fphys.2023.1321007

**Published:** 2024-01-22

**Authors:** Casper Schwartz Riedel, Birgitte Georg, Jens Hannibal

**Affiliations:** Department of Clinical Biochemistry, Faculty of Health Sciences, Bispebjerg and Frederiksberg Hospital, University of Copenhagen, Copenhagen, Denmark

**Keywords:** neuroglobin (Ngb), VIP, AVP, circadian rhythms, suprachiasmatic nucleus

## Abstract

Light-sensitive neurons are located in the ventral and central core of the suprachiasmatic nucleus (SCN), whereas stably oscillating clock neurons are found mainly in the dorsal shell. Signals between the SCN core and shell are believed to play an important role in light entrainment. Core neurons express vasoactive intestinal polypeptide (VIP), gastrin-releasing peptide (GRP), and Neuroglobin (Ngb), whereas the shell neurons express vasopressin (AVP), prokineticin 2, and the VIP type 2 (VPAC2) receptor. In rodents, light has a phase-shifting capacity at night, which induces rapid and transient expression of the EGR1 and FOS in the SCN.

**Methods:** The present study used immunohistochemical staining of FOS, EGR1, and phenotypical markers of SCN neurons (VIP, AVP, Ngb) to identify subtypes/populations of light-responsive neurons at early night.

**Results:** Double immunohistochemistry and cell counting were used to evaluate the number of SCN neurons expressing FOS and EGR1 in the SCN. The number of neurons expressing either EGR1 or FOS was higher than the total number of neurons co-storing EGR1 and FOS. Of the total number of light-responsive cells, 42% expressed only EGR1, 43% expressed only FOS, and 15% expressed both EGR1 and FOS. Light-responsive VIP neurons represented only 31% of all VIP neurons, and EGR1 represents the largest group of light-responsive VIP neurons (18%). VIP neurons expressing only FOS represented 1% of the total light-responsive VIP neurons. 81% of the Ngb neurons in the mouse SCN were light-responsive, and of these neurons expressing only EGR1 after light stimulation represented 44%, whereas 24% expressed FOS. Although most light-responsive neurons are found in the core of the SCN, 29% of the AVP neurons in the shell were light-responsive, of which 8% expressed EGR1, 10% expressed FOS, and 11% co-expressed both EGR1 and FOS after light stimulation.

**Discussion:** Our analysis revealed cell-specific differences in light responsiveness between different peptidergic and Ngb-expressing neurons in different compartments of the mouse SCN, indicating that light activates diverse neuronal networks in the SCN, some of which participate in photoentrainment.

## Introduction

In mammals, the circadian rhythms of physiology and behavior are generated from the hypothalamic suprachiasmatic nucleus (SCN), also known as the brain biological clock (Klein et al., 1991). Approximately 20,000 neurons in the SCN constitute a complex neuronal network (Abrahamson and Moore, 2001) that controls circadian rhythms due to the expression of so-called clock genes. The clock proteins drive endogenous oscillations in neuron activities, regulating internal and output signals with a period of close to but not exactly 24 h (Mohawk and Takahashi, 2011). To stay entrained with the 24 h solar cycle, the clock needs daily resetting, the most powerful signal being the light/dark cycle caused by the earth’s planetary rotation. This process, known as photoentrainment, is dependent on the inner retina and a system of intrinsically light-sensitive ganglion cells expressing the photopigment melanopsin (mRGCs) (Do and Yau, 2010; Golombek and Rosenstein, 2010; Hannibal, 2021). The mRGCs, which also receive input from the classical photoreceptors located in the outer retina (Hattar et al., 2003) send light signals to SCN neurons via the retinohypothalamic tract (RHT), a specific pathway from the mRGCs to the SCN (Hannibal, 2002), which initiates neurochemical transmission and gene expression in the SCN neurons (Hannibal, 2006; Golombek and Rosenstein, 2010). The light-responsive neurons are located in the ventral and central part of the SCN, known as the core region (Abrahamson and Moore, 2001; Morin, 2007; Fernandez et al., 2016), while rhythmically expressing neurons, primarily mediating neuronal output signals, are found in the shell region of the SCN (Foley et al., 2011; Mohawk and Takahashi, 2011). Neurons of the core and shell have been characterized by their expression of signaling molecules, receptors, and clock genes (Morin, 2007; Welsh et al., 2010; Mohawk and Takahashi, 2011). The core can be characterized by neurons expressing the neuropeptides vasoactive intestinal polypeptide (VIP), gastrin-releasing peptide (GRP), and Neuroglobin (Ngb) (Hundahl et al., 2010b; Lu and Kim, 2021), whereas the shell neurons express vasopressin (AVP), prokineticin 2 (PK2) and the VIP type 2 receptor (VPAC2) (Cheng et al., 2005; Hannibal et al., 2017; Buijs et al., 2021). In the SCN, several immediate-early genes (IEGs) encoding transcription factors are activated rapidly and transiently within minutes after light stimulation (Milbrandt, 1987; Sheng and Greenberg, 1990; Xu et al., 2021). In rodents, light stimulation has phase-shifting capacity during the night and induces a rapid and transient expression of the IEG’s *Egr1* (also called *Etr103, Krox-1, Krox-24, Krox24, Ngf1-A, Ngfi-A, NgfiA, Tis8, Zenk, Zfp-6, Zif268*) (Kilduff et al., 1998; Riedel et al., 2020) and *Fos* in the SCN (Kornhauser et al., 1996b). FOS is one of the most well-characterized markers of light-induced gene expression in the SCN (Kornhauser et al., 1990; Rusak et al., 1990; Kornhauser et al., 1996a). EGR1 is less well characterized, although extensively expressed throughout the SCN after light stimulation (Rusak et al., 1990).

We have previously shown that EGR1 is involved in light signaling mediated by the two neurotransmitters of the RHT, PACAP, and glutamate (Riedel et al., 2018; Riedel et al., 2020). However, although used in many studies, investigation of whether EGR1 and FOS are present in the same light-responsive neurons or whether signaling pathways in light-responsive neurons can be differentiated on the expression of either FOS or EGR1 or a combination (co-localization) of both genes in different subtypes of light-activated neurons have not been investigated in detail (Tanaka et al., 1997; Park et al., 2016; Xu et al., 2021). We, therefore, used a combination of FOS, EGR1, and phenotypical markers of SCN neurons (VIP, AVP, Ngb) to identify subtypes/populations of light-responsive neurons. This approach could discover different light-activated networks in the SCN, some of which participate in photoentrainment.

## Material and methods

### Animals

This study used 10 male 129/Sv mice. All animals were included in the study at 10–12 weeks of age, maintained in 12:12-h light-dark (LD) cycles in individual cages with food (Altromin 1324; Altromin Spezialfutter, Germany) and water *ad libitum*.

Animals were treated according to the principles of Laboratory Animal Care (Law on Animal Experiments in Denmark, publication 382, June 10, 1987) and under Danish Veterinary Authorities (Dyreforsoegstilsynet) license no. 2008/561-1445. The animal research ethics committee (Dyreforsoegstilsynet) granted a formal waiver of ethics approval license no: 2017-15-0201-01364 to Jens Hannibal and thereby approved the study.

### Light stimulation of animals

For light-stimulation experiments, animals received a 30 min pulse of white light (300 lux) at ZT16 in their home cages, followed by 30 min in darkness. The mice were killed in dim red light (<3 lux) and fixated 60 min after the initiation of light exposure by perfusion through the heart with Stefanini’s fixative (2% PFA, 15% picric acid in 0.1 M PBS, pH 7.2). After removal, the brain was immersion fixated in the same fixative overnight. Light intensity was measured using an Advantest Optical Power meter TQ8210 (MetricTest, Hayward, CA), and measurements were determined at settings of 514 nm, 300 lux (115 μW/cm2).

### Double and triple-antigen immunohistochemistry

The brains were cut on a cryostat in 40-μm-thick free-flowing coronal sections, as described previously (Hannibal et al., 2017), and stored at −21°C in cryoprotectant. All brains were processed as free-floating sections for fluorescence immunohistochemistry (IHC). IHC was performed as previously described (Hannibal et al., 2014). Briefly, tissue was rinsed with 0.25% Triton-X-100 (TX) in 0.01 M PBS between all steps of the procedure, and all incubations included 0.25% TX and 0.25% bovine serum albumin (BSA). All rinses and incubations occurred at room temperature unless otherwise noted. Before staining, the tissue was post-fixed in 4% paraformaldehyde for 120 min at room temperature, followed by pre-incubation in 1% H_2_O_2_ in 0.01 M PBS for 10 min, blocked with 5% donkey serum for 20 min, and then incubated with rabbit EGR1 antibody (diluted 1:10.000) (C-19; code no: SC-189, Santa Cruz Biotechnology, Inc.) overnight at 4°C. The EGR1 antibody was visualized by a biotinylated donkey anti-rabbit antibody (code no: 711-065-152 Jackson Immunoresearch Laboratories, Baltimore, PA, United States, diluted 1:500 and incubated overnight at 4°C) in combination with Avidin-Biotin-peroxidase Complex (ABC) (VWR International, Roedovre Denmark), followed by biotinylated tyramide (Tyramide System Amplification, PerkinElmer Waltham, MA, United States) and streptavidin-Alexa488 (code no: 016-540-084 Jackson Immunoresearch Laboratories, Baltimore, PA, United States, diluted 1:500). Hereafter, tissue was washed in PBS and incubated with rabbit anti-FOS antiserum (code 9412-6, kindly donated by Dr. P.J. Larsen) raised against amino acids 4–17 of the human/rat proteins (Woldbye et al., 1996) (diluted 1:1,000) or rabbit-anti Ngb (characterized previously in Hannibal et al. (2011), and obtained from Dr. Sylvia Dewilde, University of Antwerpen, (Hundahl et al., 2010b), diluted 1:20.000) overnight at 4°C. On the third day, both FOS and Ngb were visualized by incubation with Alexa-594 donkey anti-rabbit A21207 (Molecular probes, Life Technologies, United States, diluted 1:800). For triple IHC a third antibody was added together with the anti-FOS antiserum in the last step. Briefly, VIP B-GP-340-1 (EuroDiagnostica, Malmo, Sweden, diluted 1:1500) or AVP (GHC 8103) (Guinea pig, polyclonal anti AVP, Peninsula Laboratories, CA. Code: T-5048, diluted 1:500) were added and incubated overnight at 4°C. Finally, both VIP and AVP were visualized by Alexa649 donkey anti-guinea-pig 706-495-148 (Molecular probes, Life Technologies, United States, diluted 1:200).

### Photomicrographs

Fluorescent images were obtained using an iMIC confocal microscope (Till Photonics, FEI, Germany) equipped with appropriate filter settings for detecting DAPI, Cy2/Alexa Fluor 488, Texas Red/AlexaFluor561/594, and Cy5/Alexa640. The iMIC confocal microscope was equipped with the following objectives: ×10, numerical aperture (NA) = 0.35; X20, NA = 0.75; X40, NA = 1.3 and X60, NA = 1.46. Using the X60, the highest resolution ((r = λ/NA), where λ is the imaging wavelength) was for X60 = 174 nm. Resolution in the z-axis was at X60 0.2 µm. All images used for cell counting and illustrations were photographed through the 40 µm thickness of each section. Z-stacks photographed using the X40 or ×60 objective were deconvoluted in AutoQuantX, version 3.04 (Media Cybernetics, Inc. Rockville, United States) before being analyzed in Fiji or IMARIS^®^ vers. 10,0.1 (RRID:SCR_007370) from Bitplane, Switzerland (http://www.bitplane.com). 3D-reconstruction performed in IMARIS^®^. All images were adjusted for brightness and contrast in Fiji or Photoshop CS5 (Adobe, San Jose, CA, RRID:SCR_014199) and mounted into plates in Adobe Illustrator CS5 (Adobe).

### Quantification of EGR1 and FOS-positive cells

EGR1 and FOS positive cells and co-localization of EGR1 and FOS were quantified in sections from three animals' rostral,—mid, and caudal SCN (Figures 5A, B; Supplementary Table S1). The same three levels of the SCN in two animals (Figures 5C–E; Supplementary Tables S2, S3) were analyzed regarding the expression of the phenotypical markers (VIP, AVP and Ngb). In each mouse, cells were manually counted bilateral in a section from the rostral, mid, and caudal parts of the SCN, respectively. Ngb is primarily found in the rostral and mid portion of the SCN (Figures 3C, G), and only sections from these two parts of the SCN were counted for Ngb-positive neurons. All cell counting was done using the cell counter plugin in Fiji software (version 1.47q, NIH, United States) on the entire Z-stack of 40 μm from each level of the SCN section. Examples of co-localization (Ngb, EGR1, and FOS) were also presented in 3D using the co-localization module of IMARIS^®^ (Figure 4).

## Results

### Light-induced EGR1 and FOS-expression in the mouse SCN

**FIGURE 1:**
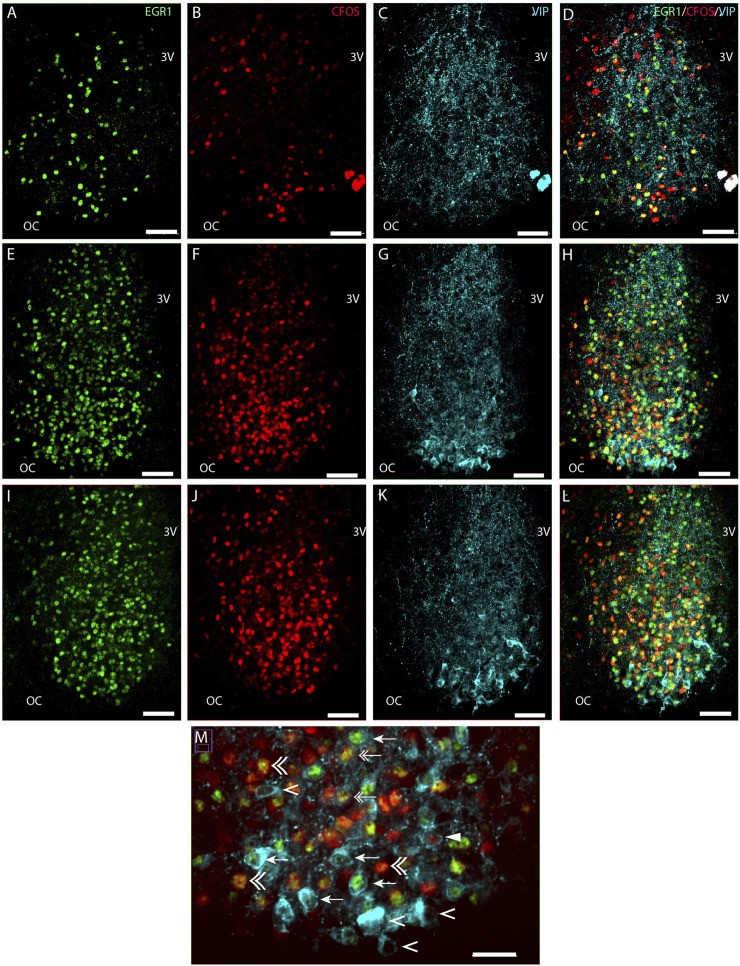
Light-induced EGR1 and FOS expression in VIP expressing neurons of the mouse SCN ZT17. Mice were light stimulated early night (ZT16), and expression of EGR1 and FOS at ZT17 was visualized by immunohistochemistry. The panels in the first column shows EGR1 (green) expression in sections of rostral **(A)**, mid **(E)**, and caudal **(I)** SCN. In the second column light-induced FOS (red) expression in sections of rostral **(B)**, mid **(F)**, and caudal **(J)** SCN are shown. The third column shows the expression of VIP neurons (light blue) in sections of rostral **(C)**, mid **(G)**, and caudal **(K)** SCN. The fourth column shows merged images of EGR1/FOS/VIP in sections of rostral **(D)**, mid **(H)**, and caudal **(L)** SCN. The ventral part in the mid-section of SCN is shown in higher magnification in panel M. Co-localization of EGR1 and VIP are indicated by arrows, VIP and FOS by solid arrowheads, both EGR1 and FOS in VIP by double arrows, VIP with neither EGR1 nor FOS expression by single arrowheads and EGR1 and FOS co-expression outside VIP neurons by double arrowhead. OC; optic chiasm; 3V, third ventricle. Scale bar 50 µm **(A–L)** and 25 µm **(M)**.

**FIGURE 2:**
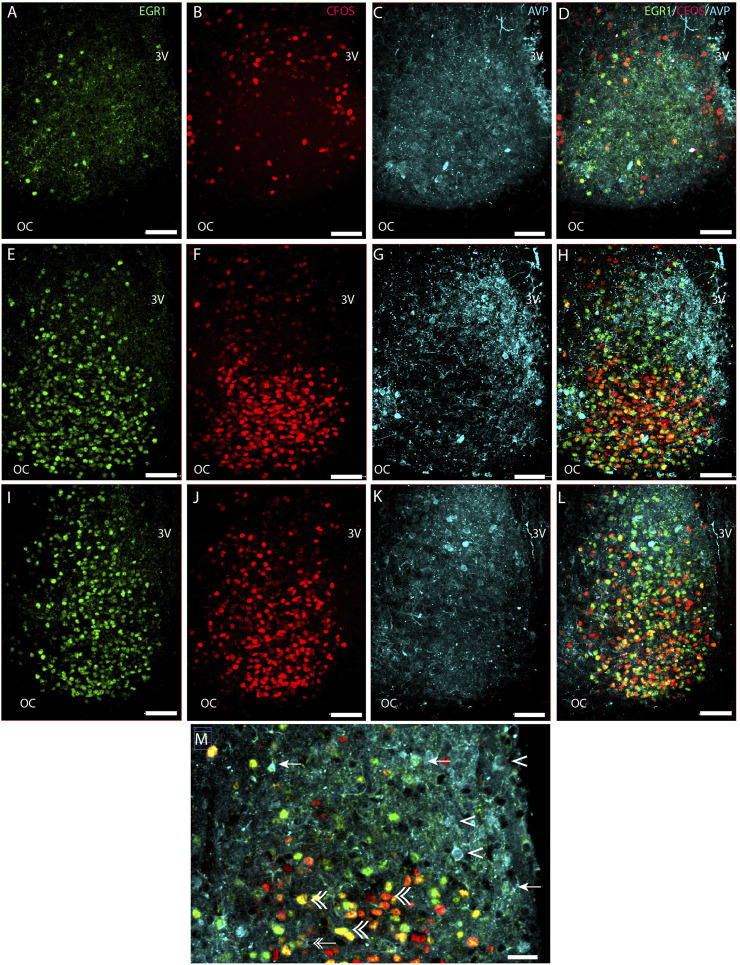
Light-induced EGR1 and FOS expression in AVP-expressing neurons of the mouse SCN. The panels in the first column shows light-induced EGR1 (green) expression in sections of rostral **(A)**, mid **(E)**, and caudal **(I)** SCN. The panels in the second column shows the FOS (red) expression in sections of rostral **(B)**, mid **(F)**, and caudal **(J)** SCN, and in the third column, the expression of AVP neurons (light blue) in sections of rostral **(C)**, mid **(G)**, and caudal **(K)** SCN are shown. The fourth column shows merged images of EGR1/FOS/AVP in sections of rostral **(D)**, mid **(H)**, and caudal **(L)** SCN. Higher magnification of the ventral part in the mid-section of SCN **(M)** shows co-localization of EGR1 and AVP (arrows). Double arrows show examples of both EGR1 and FOS in AVP cells, AVP with neither EGR1 nor FOS expression by single arrowhead, and EGR1 and FOS co-expression outside AVP neurons by double arrowhead. OC; optic chiasm; 3V, third ventricle. Scale bar 50 µm **(A–L)** and 25 µm **(M)**.

In all light-stimulated animals examined, EGR1 and FOS-immunoreactivity (IR) were found throughout the rostro-caudal extent of the SCN, with the core region more densely stained in sections from the mid and caudal part of the SCN (Figures 1A, E, I; Figures 2A, E, I). Previous studies have shown that only a few cells positive for EGR1 or FOS IR are present in the SCN of animals taken at the same time but without previous light stimuli (Hundahl et al., 2012; Riedel et al., 2020). Of the total number of light-responsive cells, 42% expressed only EGR1, 43% expressed only FOS, and 15% expressed both EGR1 and FOS (Figure 5A, Supplementary Table S1). The distribution of light responsive neurons in the rostral, mid–and caudal SCN are shown in Figure 5B and Supplementary Table S1. Of 1418 light-responsive neurons in the rostral SCN, 38% express EGR1, and 47% express FOS and 15% expressed both EGR1 and FOS (Figure 1A; Figure 2A; Figure 5B) (Supplementary Table S1). The mid-SCN had the highest density of both EGR1 and FOS expressing cells (total cell count 5455 neurons), and 42% of these were EGR1 positive neurons, while 44% expressed FOS and 14% expressed both EGR1 and FOS (Figure 5B) (Supplementary Table S1). 41% of 4308 light-responsive neurons in the caudal SCN expressed EGR1, and 42% expressed FOS and 17% expressed both EGR1 and FOS (Figure 1; Figure 2; Figure 5B) (Supplementary Table S1). At all three levels of the SCN, the number of neurons storing either solely EGR1 or FOS was higher than the number of neurons co-storing EGR1 and FOS (Figures 5A, B).

### Light-induced EGR1-and FOS-expression in VIP neurons in the mouse SCN

We next investigated the distribution of light-induced EGR1 and/or FOS in VIP-containing neurons in the ventral SCN. VIP neurons were found in the core SCN, mainly in the ventral part (Figures 1C, G, K). Of 741 counted VIP neurons, light-responsive VIP neurons represented only 31% of all VIP neurons, and EGR1 represents the largest group of light-responsive VIP neurons (18%). VIP neurons expressing only FOS represented less than 1% of the total light-responsive VIP neurons (Figures 5A, C and Supplementary Table S2). The distribution of light responsive VIP neurons in the rostral, mid–and caudal SCN are shown in Figure 5C and Supplementary Table S2 and show that most of the light responsive VIP neurons are found in the mid and caudal SCN.

### Light-induced EGR1-and FOS-expression in AVP neurons in the mouse SCN

Next, we investigated whether the light-activated neurons could also be detected in the AVP neuron population located in the shell region of the SCN (Abrahamson and Moore, 2001) (Figure 2). Although most light-responsive neurons are found in the core of the SCN, we found that of 554 AVP cells 29% of the AVP neurons in the shell were light-responsive, of which 8% expressed EGR1, 10% expressed FOS, and 11% co-expressed both EGR1 and FOS after light stimulation. (Figures 2D, H, L, M; Figures 5A, D; Supplementary Table S3). The distribution of light responsive AVP neurons in the rostral, mid–and caudal SCN are shown in Figure 5D and Supplementary Table S3 and demonstrate that most of the light response AVP neurons are located in the caudal SCN (Figure 5D).

### Light-induced EGR1 and FOS-expression in Ngb neurons in the mouse SCN

**FIGURE 3:**
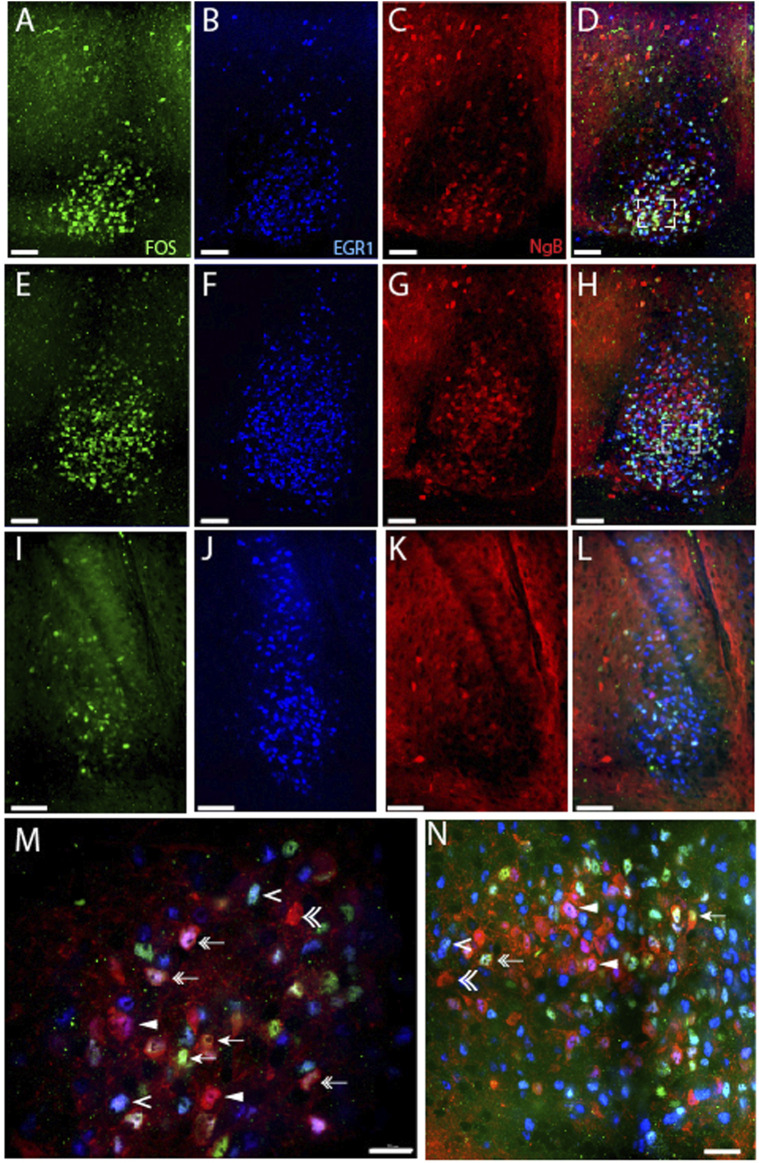
Light-induced EGR1 and FOS expression in Ngb expressing neurons of the mouse SCN. The panels in the first column shows light-induced FOS (green) expression in sections of rostral **(A)**, mid **(E)**, and caudal **(I)** SCN at ZT 17 after light stimuli early night (ZT16). The second column shows EGR1 expressing neurons (blue) in sections of rostral **(B)**, mid **(F)**, and caudal **(J)** SCN. The third column shows the expression of Ngb neurons (red) in sections of rostral **(C)**, mid **(G)**, and caudal **(K)** SCN. The fourth column shows merged images of FOS/EGR1/Ngb stainings in sections of rostral **(D)**, mid **(H)**, and caudal **(L)** SCN. Higher magnification of the rostral **(M)** and mid-section of SCN **(N)** shows examples of co-localization of FOS/Ngb (arrows), both FOS and EGR1 in Ngb expressing neurons (double arrow), Ngb and only EGR1 (solid arrowhead), Ngb with neither EGR1 nor FOS expression (double arrowhead), and EGR1 and FOS co-expression in Ngb negative neurons (single arrowhead). OC; optic chiasm; 3V, third ventricle. Scale bars 50 µm **(A–L)** and 20 µm **(M, N)**.

**FIGURE 4:**
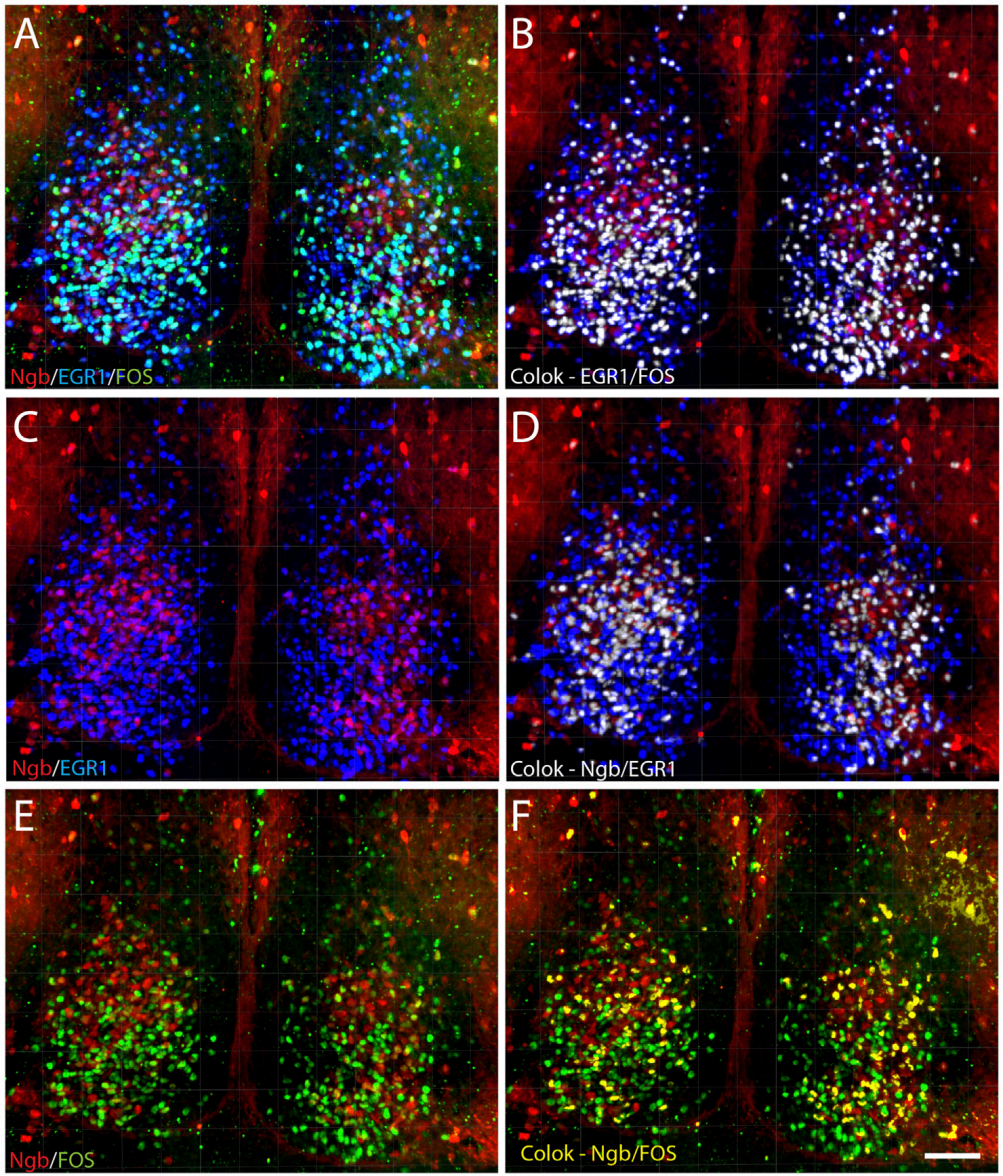
Distribution of light-induced neurons in the mid-SCN expressing Ngb, EGR1, and FOS visualized after 3D analysis. 3D reconstruction of a section of the mid-SCN containing the highest concentration of Ngb neurons is visualized after light-induced expression of EGR1 and FOS **(A)** followed by computer-based co-localization (pixel overlap) of EGR1 and FOS (white in **B**), only Ngb and EGR1 **(C)** and co-localizing neurons (white in **D**) and co-localization of Ngb and FOS (yellow in **F**). Scale bar 50 µm **(A–F)**.

**FIGURE 5:**
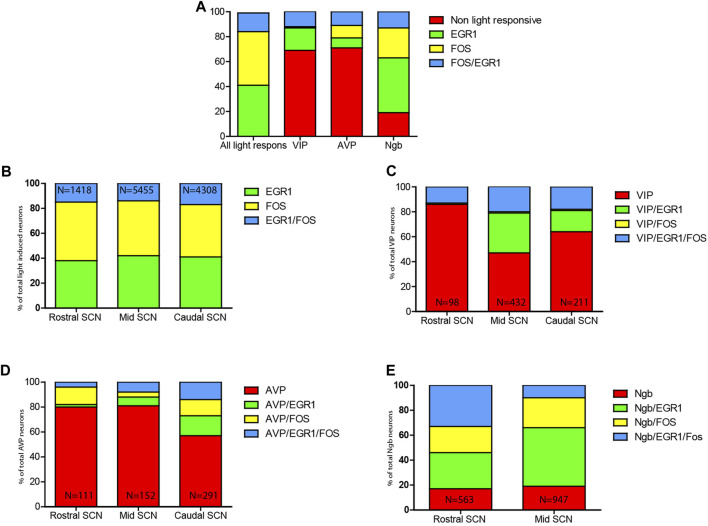
Distribution of light-induced neurons in the mouse SCN. **(A)**. Relative distribution of EGR1 and FOS and EGR1 and FOS in the mouse SCN (lane 1) followed by the relative number of VIP neurons (lane 2) being light responsive evaluated by the expression of either EGR1, FOS or EGR1and FOS. In lane 3 the relative number of AVP neurons being light responsive as evaluated by the expression of either EGR1, FOS or EGR1 and FOS is shown followed by lane 4 showing the relative number of Ngb neurons being light responsive as evaluated by the expression of either EGR1, FOS or EGR1 and FOS. **(B)**. Relative numbers of SCN neurons expressing either EGR1, FOS, or EGR1 and FOS (N numbers indicate the total number of neurons counted in the rostral, mid, and caudal SCN). **(C)**. Relative numbers of VIP SCN neurons expressing either VIP/EGR1, VIP/FOS, or VIP/EGR1/FOS or VIP (not light responsive). **(D)**. Relative numbers of AVP SCN neurons expressing either AVP/EGR1, - AVP/FOS or AVP/EGR1/FOS or AVP (not light responsive). **(E)**. Relative numbers of Ngb SCN neurons expressing either Ngb/EGR1, - Ngb/FOS or Ngb/EGR1/FOS or Ngb (not light responsive) (N numbers indicate the total number of neurons (VIP, AVP or Ngb) counted in the rostral, mid, and caudal SCN). See also Supplementary Tables S1-S4.

The largest group of neurons expressing Ngb are located in the rostral and central core of the mouse SCN (Hundahl et al., 2010a), with very few Ngb neurons located in the caudal SCN (Figures 3C, G, K). 81% of Ngb neurons in the mouse SCN were light-responsive, and of these 44% express only EGR1 after light stimulation whereas 24% expressed FOS (Figure 5A). The distribution of light responsive Ngb neurons in the rostral and mid SCN are shown in Figure 5E and Supplementary Table S4 and demonstrate that the Ngb neurons expressing only EGR1 represent the largest group of Ngb neurons in the mid SCN (Figures 4, 5E; Supplementary Table S4).

## Discussion

The present study was undertaken to characterize the light-induced neurons in the mouse SCN based on the induction of two light-responsive genes, *Egr1* and *Fos* (Xu et al., 2021). These markers were used to investigate whether different populations of light-induced neurons could be differentiated due to expression of phenotype markers, such as VIP, Ngb, and AVP, located in three different compartments of the SCN, namely the ventral core, central core, and shell, respectively. Our analysis revealed cell-specific differences in light responsiveness between different peptidergic and Ngb-expressing neurons in the different compartments of the mouse SCN, indicating that light activates different neuronal networks in the SCN, some of which participate in photoentrainment.

### Light activation of VIP neurons in the SCN

VIP neurons are located in the retino-recipient part of the ventral core of the mouse SCN, which receives retinal input from the melanopsin-containing ganglion cells of the RHT (Hannibal et al., 2017). Previous studies have shown that VIP neurons respond to light by induction of FOS (Romijn et al., 1996; Silver et al., 1996; Hannibal and Fahrenkrug, 2004). However, the fraction of light-responsive VIP cells has not been addressed in detail (Park et al., 2016; Xu et al., 2021). The present study demonstrates that VIP neurons in the ventral core primarily express EGR1 or both EGR1 and FOS, while only less than 1% of the VIP neurons in the mouse respond to light stimulation by induction of only FOS. FOS has previously been demonstrated in VIP neurons in the SCN in rats and hamsters (Romijn et al., 1996; Silver et al., 1996; Hannibal and Fahrenkrug, 2004) but not in combination with EGR1. In a recent study using bulk RNA-seq and single-nucleus RNA-seq of SCNs, Xu et al. (2021) analyzed clusters of neurons in the SCN after light stimuli and demonstrated that VIP-containing clusters were found to express FOS, EGR1, and NPAS4. However, the concomitant expression of these IEGs in single neurons was not reported (Xu et al., 2021). Another study using single-cell RNA clustering of subtypes of SCN cells identified VIP-expressing neurons co-expressing Neuromedin S (NMS) or GRP (Wen et al., 2020), indicating that neurons expressing VIP represent a heterogenous group, some of which are light-responsive and some are not (Todd et al., 2020; Wen et al., 2020; Xu et al., 2021). Accordingly, we found that VIP-expressing neurons are heterogeneous in their responsiveness to light as evaluated by the expression of FOS and EGR1 or both. The study by Wen et al. (Wen et al.), confirmed that VIP/GRP clusters express the PACAP type 1 receptor (Wen et al., 2020), the target for RHT nerve terminals, as we reported previously (Hannibal et al., 2017). The findings support an important role of VIP in light entrainment (Jones et al., 2018; Todd et al., 2020) and demonstrate the complexity of SCN networks, where VIP neurons in the ventral SCN interact with diverse neuronal populations (Ono et al., 2021). Furthermore, VIP also projects internally within the SCN (LeSauter et al., 2002; Hannibal et al., 2019), activating neurons in the shell via the VPAC2 receptor (Vosko et al., 2015; Hannibal et al., 2017; Hamnett et al., 2021). This projection is essential for the synchronization of SCN neurons and is necessary for the overall stable rhythmicity of SCN-regulated circadian rhythms (Maywood et al., 2011; Vosko et al., 2015; Hannibal et al., 2017; Todd et al., 2020).

Other SCN neurons project externally to the anterior and mid-and dorsal hypothalamus, representing output-regulating nerve fibers, being involved in the circadian regulation of physiological rhythms (Paul et al., 2020; Ono et al., 2021) such as the HPA- and HPG axis via the VPAC1 and the VPAC2 receptor (Stangerup and Hannibal, 2020). It is likely that signaling pathways initiated by the induction of either EGR1 or FOS or both couple to different functionalities.

### Light activation of AVP-expressing neurons in the SCN

Approximately 20% of the SCN neurons express AVP, most of which are located in the dorsomedial SCN (Abrahamson and Moore, 2001). AVP is considered a weaker synchronizer of SCN rhythmicity compared to VIP. This knowledge is based on the selective elimination of the core clock gene *Bmal1/Arntl* in AVP neurons, which have limited influence on circadian-driven running wheel activity (Mieda et al., 2015). Although AVP neurons are not considered direct light-responsive due to their location in the shell (Antle and Silver, 2005), our study demonstrates that 29% of AVP neurons, most of these located in the caudal part of the SCN shell, co-stored either EGR1, FOS, or both after light stimulation at early night. AVP is a clock-controlled gene with strong circadian oscillation peaking during the subjective day (Jin et al., 1999; Yamaguchi et al., 2013). Thus, it is possible that the number of light-responsive AVP neurons in the present study could be underestimated.

It remains to be shown whether AVP neurons in the SCN are directly innervated from the eyes or indirectly via light-sensitive VIP neurons in the ventral SCN projecting to the dorsal shell region (Abrahamson and Moore, 2001; Stangerup and Hannibal, 2020), where the VPAC2 receptor is found on neurons (Hannibal et al., 2017; Hannibal et al., 2019), some of which express AVP (An et al., 2012). AVP signaling in the SCN is via the V1a and V1b receptors (Bedont et al., 2018) and https://mouse.brain-map.org/gene/show/33433 and https://mouse.brain-map.org/gene/show/26109. Interestingly, mice lacking both types of AVP receptors (V1a^−/−^V1b^−/−^) have a significantly altered response to a large (8h) phase advance of the LD cycle (Yamaguchi et al., 2013). Recently, Bedont et al. (Bedont et al., 2018) provided evidence that AVP signaling via the V1a and V1b receptors participates in regulating both period length and phase. Therefore, light activation of AVP neurons via EGR1 and FOS could plays a role in SCN intrinsic resistance to external perturbation (light) via signaling through the V1a and V1b receptors.

### Light activation of Ngb-expressing neurons in the SCN

We have previously characterized the localization and expression of Ngb in rats (Hundahl et al., 2010b) and mouse SCN (Hundahl et al., 2012). In the rat SCN, a subpopulation of Ngb neurons, some of which co-stored GRP, was found to express FOS after light stimulation at night. In the mouse, light-induced phase delay at early night involves a mechanism dependent on Ngb since Ngb-deficient mice show larger phase delays compared to wild-type mice despite having a clock-controlled free-running rhythm with a period length as wild-type mice (Hundahl et al., 2012). Using EGR1 in combination with FOS, we now identified more light-responsive Ngb-positive neurons than previously, where only FOS was used as a marker. However, the functional role of Ngb in core-located neurons of the mouse SCN is not clear. In both rats and mice, Ngb neurons receive neuronal inputs from RHT fibers from the eyes but also from NPY-containing nerve fibers of the intergeniculate leaflet (IGL) (Hundahl et al., 2010b; Hundahl et al., 2012). Neuronal inputs from the IGL represent so-called non-photic information which modulated light responses in SCN neurons (Yannielli and Harrington, 2000; 2001). The localization of Ngb neurons in the retinorecipient part of the SCN which also receives non-photic input from the IGL and the medial raphe (Hannibal and Fahrenkrug, 2006; Morin, 2013), makes it possible that these neurons use different signaling pathways such as EGR1, FOS or both, which although not well understood could act as a hub for external signal integration and phase shaping of the internal clock driven rhythm of the SCN.

## Conclusion

Our analysis revealed cell-specific differences in light responsiveness between different peptidergic and Ngb-expressing neurons in the compartments of the mouse SCN. The study extends previous studies indicating that light activates various neuronal networks in the SCN, some of which participate in photoentrainment.

## Data Availability

The raw data supporting the conclusion of this article will be made available by the authors, without undue reservation.
