# Supplementary material for: Phenotyping of light-activated neurons in the mouse SCN based on the expression of FOS and EGR1
Source: Front Physiol. 2024 Jan 22;14:1321007. doi: 10.3389/fphys.2023.1321007 (PMC10839010; doi:10.3389/fphys.2023.1321007)
Supplement: Supplementary file 1 [file Table1.docx]

Supplementary Table 1 Total EGR1 and FOS cells in the SCN ZT17 and co-expression

| Animal 1 | SCN | EGR1 | FOS | EGR1+FOS | Total |
| --- | --- | --- | --- | --- | --- |
|  | Rostral | 162 | 179 | 62 | 403 |
|  | Mid | 695 | 648 | 167 | 1510 |
|  | Caudal | 498 | 560 | 172 | 1230 |
|  | Total | 1355 | 1387 | 401 | 3143 |
|  |  | **43%** | **44%** | **13%** |  |
|  |  |  |  |  |  |
| Animal 2 | SCN | EGR1 | FOS | EGR1+FOS | Total |
|  | Rostral | 69 | 231 | 40 | 340 |
|  | Mid | 628 | 660 | 176 | 1464 |
|  | Caudal | 806 | 827 | 338 | 1971 |
|  | Total | 1503 | 1718 | 554 | 3775 |
|  |  | **40%** | **46%** | **15%** |  |
|  |  |  |  |  |  |
| Animal 3 | SCN | EGR1 | FOS | EGR1+FOS | Total |
|  | Rostral | 296 | 265 | 114 | 675 |
|  | Mid | 994 | 1063 | 424 | 2481 |
|  | Caudal | 483 | 429 | 195 | 1107 |
|  | Total | 1773 | 1757 | 733 | 4263 |
|  |  | **42%** | **41%** | **17%** |  |
|  |  |  |  |  |  |
| **Average of all animals**  **% of all light-responsive cells** | | **1544** | **1621** | **563** | **3727** |
|  |  | **42%** | **43%** | **15%** |  |

Supplementary Table 2 EGR1, FOS and co-expression in VIP cells

| Animal 1 | SCN | EGR1+VIP | FOS+VIP | EGR1+FOS+  VIP | VIP only | VIP total |
| --- | --- | --- | --- | --- | --- | --- |
|  | Rostral | 1 | 0 | 0 | 11 | 12 |
|  | Mid | 52 | 1 | 19 | 149 | 221 |
|  | Caudal | 20 | 2 | 21 | 120 | 163 |
|  | Total | 73 | 3 | 40 | 280 | 396 |
|  |  | **18%** | **1%** | **10%** | **71%** |  |
|  |  |  |  |  |  |  |
| Animal 2 | SCN | EGR1+VIP | FOS+VIP | EGR1+FOS+ VIP | VIP only | VIP total |
|  | Rostral | 16 | 0 | 9 | 61 | 86 |
|  | Mid | 39 | 0 | 40 | 132 | 211 |
|  | Caudal | 6 | 0 | 6 | 36 | 48 |
|  | Total | 61 | 0 | 55 | 229 | 345 |
|  |  | **18%** | **0%** | **16%** | **66%** |  |
|  |  |  |  |  |  |  |
| **Total of all animals** | | **134** | **3** | **95** | **509** | **741** |
| **% of light-responsive VIP cells of total VIP cells** | | **18%** | **< 1%** | **13%** | **69%** |  |

Supplementary Table 3 EGR1, FOS and co-expression in AVP cells

| Animal 1 | SCN | EGR1+AVP | FOS+AVP | EGR1+FOS  + AVP | AVP only | AVP total |
| --- | --- | --- | --- | --- | --- | --- |
|  | Rostral | 3 | 12 | 4 | 19 | 38 |
|  | Mid | 7 | 2 | 4 | 89 | 102 |
|  | Caudal | 27 | 13 | 25 | 140 | 205 |
|  | Total | 37 | 27 | 33 | 248 | 345 |
|  |  | **11%** | **8%** | **10%** | **72%** |  |
|  |  |  |  |  |  |  |
| Animal 2 | SCN | EGR1+AVP | FOS+AVP | EGR1+FOS  + AVP | AVP only | AVP total |
|  | Rostral | 1 | 12 | 3 | 57 | 73 |
|  | Mid | 2 | 3 | 6 | 39 | 50 |
|  | Caudal | 4 | 12 | 20 | 50 | 86 |
|  | Total | 7 | 27 | 29 | 146 | 209 |
|  |  | **3%** | **13%** | **14%** | **70%** |  |
|  |  |  |  |  |  |  |
| **Total of all animals** |  | **44** | **54** | **62** | **394** | **554** |
| **% of light-responsive AVP of total AVP cells** | | **8%** | **10%** | **11%** | **71%** |  |

Supplementary Table 4 EGR1 and FOS neurons co-expressing Ngb

| Animal 1 | SCN | EGR1+Ngb | FOS+Ngb | EGR1+FOS  + Ngb | Ngb only | Ngb total |
| --- | --- | --- | --- | --- | --- | --- |
|  | Rostral | 72 | 54 | 39 | 18 | 183 |
|  | Mid | 356 | 146 | 64 | 58 | 624 |
|  | Total | 428 | 200 | 103 | 76 | 807 |
|  |  | **53%** | **25%** | **13%** | **9%** |  |
|  |  |  |  |  |  |  |
| Animal 2 | SCN | EGR1+Ngb | FOS+Ngb | EGR1+FOS  + Ngb | Ngb only | Ngb total |
|  | Rostral | 138 | 88 | 71 | 83 | 380 |
|  | Mid | 91 | 79 | 29 | 124 | 323 |
|  | Total | 229 | 167 | 100 | 207 | 703 |
|  |  | **33%** | **24%** | **14%** | **29%** |  |
|  |  |  |  |  |  |  |
| **Total of all animals** |  | **657** | **367** | **203** | **283** | **1510** |
| **% of light-responsive Ngb cells of total Ngb cells** | | **44%** | **24%** | **13%** | **19%** |  |
